# Supplementary material for: The Role of Bronchoalveolar Lavage in Systemic Sclerosis Interstitial Lung Disease: A Systematic Literature Review
Source: Pharmaceuticals (Basel). 2022 Dec 19;15(12):1584. doi: 10.3390/ph15121584 (PMC9781787; doi:10.3390/ph15121584)
Supplement: Supplementary file 1 [file pharmaceuticals-15-01584-s001.zip › SUPPLEMENTARY DATA S1_PEO.pdf]

SUPPLEMENTARY DATA S1

Data S1. Patients exposure outcome (PEO) framework for eligibility of research question.

The ***PEO*** *question format is used for qualitative research questions. Questions based on this format identify three concepts: (1)* ***P****opulation, (2)* ***E****xposure, and (3)* ***O****utcome(s).*

Questions are outlined below:

- In Systemic Sclerosis (SSc) patients (P), which is the prevalence (O) of BAL execution (E)?
- In Systemic Sclerosis (SSc) patients with secondary interstitial lung disease (ILD) (P), which is the prevalence (O) of BAL execution (E)?
- Which diagnostic criteria (O) were used in SSc-ILD patients (P), to define the presence of alveolitis at BAL (E)?
- In SSc-ILD patients (P), which is the prevalence (O) of alveolitis features at BAL (E)?
- In SSc-ILD patients (P), the presence of positive BAL (E) determines lung functional test alteration (O)?
- In SSc-ILD patients (P), the presence of positive BAL(E) determines FVC or FEV1 decrease (O)?
- In SSc-ILD patients (P), the presence of positive BAL (E) determines DLCO decrease (O)?
- In SSc-ILD patients (P), the presence of positive BAL (E) is associated to previous immunosuppressive treatment (O)?
- In SSc-ILD patients (P), the presence of positive BAL (E) determines worsening on prognosis (O)?
- In SSc-ILD patients (P), the presence of positive BAL (E) determines radiological progression of lesions in follow up (O)?
- In SSc-ILD patients (P), the presence of positive BAL (E) determines clinical worsening (O)?
- In SSc-ILD patients (P), the presence of positive BAL (E) determines progressive functional deterioration (O)?
- In SSc-ILD patients (P), the presence of positive BAL (E) determines death for respiratory failure (O)?
- In SSc-ILD patients (P), the presence of positive BAL (E) is treated with specific therapy (O)?
- In SSc-ILD patients (P), the presence of positive BAL (E) is treated with immunosuppressive therapy (O)?
