# Supplementary material for: The Role of Bronchoalveolar Lavage in Systemic Sclerosis Interstitial Lung Disease: A Systematic Literature Review
Source: Pharmaceuticals (Basel). 2022 Dec 19;15(12):1584. doi: 10.3390/ph15121584 (PMC9781787; doi:10.3390/ph15121584)
Supplement: Supplementary file 1 [file pharmaceuticals-15-01584-s001.zip › SUPPLEMENTARY TABLE S1_QUADAS.pdf]

SUPPLEMENTARY TABLE S1

Table S1. Quality assessment of diagnostic accuracy studies (QUADAS) for articles included in the systematic review.

	bias patient selection	bias index test	bias reference standard	bias flow & timing	applicability patient selection	applicability index test	applicability reference standard
Bouros et al (19)	0	0	0	0	0	0	0
Cailes et al (20)	0	0	2	0	0	0	0
Clements et al (21)	0	0	0	0	0	0	0
De Santis et al (22)	0	0	0	0	0	0	0
Goh et al (23)	2	0	0	0	0	2	0
Goldin et al (24)	0	0	0	0	0	0	0
Hant et al (25)	0	0	0	0	0	0	0
Kowal-Bielecka et al (26)	0	0	0	0	0	0	0
Moodley et al (27)	0	0	0	0	0	0	0
Nagasawa et al (28)	0	0	0	0	0	0	0
Prasse et al (29)		0	0	0	0	0	0
Salaffi et al (30)		0	0	0	0	0	0
Schmidt et al (31)	0	0	0	0	0	0	0
Southcott et al (32)	0	0	0	0	0	0	0
Volpinari et al (33)	2	0	0	0	0	0	0
Yilmaz et al (34)	2	0	0	0	2	0	0
Wells et al (35)	0	0	0	0	0	0	0
Behr et al (36)	0	0	0	0	0	0	0

QUADAS-2 comprises 4 domains: patient selection, index test, reference standard, and flow and timing. Each domain is assessed in terms of risk of bias; and the patient selection, index test, and reference standard domains are also assessed regarding applicability. 0= low risk; 1= high risk; 2= unclear risk.
